# Human neuronal cell protein responses to Nipah virus infection

**DOI:** 10.1186/1743-422X-4-54

**Published:** 2007-06-07

**Authors:** Li-Yen Chang, AR Mohd Ali, Sharifah Syed Hassan, Sazaly AbuBakar

**Affiliations:** 1 Center for Proteomics Research, Department of Forest Biotechnology, Forest Research Institute Malaysia, 52109, Selangor, Malaysia; 2 Veterinary Research Institute, Jalan Sultan Azlan Shah, 13800 Ipoh, Perak, Malaysia; 3 Department of Medical Microbiology, Faculty of Medicine, University Malaya, 50603, Kuala Lumpur, Malaysia

## Abstract

**Background:**

Nipah virus (NiV), a recently discovered zoonotic virus infects and replicates in several human cell types. Its replication in human neuronal cells, however, is less efficient in comparison to other fully susceptible cells. In the present study, the SK-N-MC human neuronal cell protein response to NiV infection is examined using proteomic approaches.

**Results:**

Method for separation of the NiV-infected human neuronal cell proteins using two-dimensional polyacrylamide gel electrophoresis (2D-PAGE) was established. At least 800 protein spots were resolved of which seven were unique, six were significantly up-regulated and eight were significantly down-regulated. Six of these altered proteins were identified using mass spectrometry (MS) and confirmed using MS/MS. The heterogenous nuclear ribonucleoprotein (hnRNP) F, guanine nucleotide binding protein (G protein), voltage-dependent anion channel 2 (VDAC2) and cytochrome bc1 were present in abundance in the NiV-infected SK-N-MC cells in contrast to hnRNPs H and H2 that were significantly down-regulated.

**Conclusion:**

Several human neuronal cell proteins that are differentially expressed following NiV infection are identified. The proteins are associated with various cellular functions and their abundance reflects their significance in the cytopathologic responses to the infection and the regulation of NiV replication. The potential importance of the ratio of hnRNP F, and hnRNPs H and H2 in regulation of NiV replication, the association of the mitochondrial protein with the cytopathologic responses to the infection and induction of apoptosis are highlighted.

## Background

Nipah virus (NiV) is a recently discovered zoonotic negative-stranded RNA virus of the genus *Henipavirus *of the *Paramyxoviridae *family [1,2]. The virus causes severe to fatal central nervous system (CNS) infection in humans [3,4]. The virus is acquired from contact with the excretions or secretion of NiV-infected pigs [5-7] and it has a mortality rate of ~40% in human infection. NiV-infected patients typically present with symptoms of CNS infection with elevated cerebrospinal fluid protein and white cell counts [6]. Severe vasculitis and small lesions with presence of NiV antigen and nucleocapsid inclusion bodies are also detectable in the brain using immunohistochemical staining [8,9], but no mature viral particles are observed [10,11]. NiV productively infects several different human cell types and cells of other host origin [12]. In contrast to infections of the fully susceptible human lung fibroblast and pig kidney cells, NiV replicates less efficiently in human neuronal cells. It does not result in immediate cell lysis and releases low number of infectious virus particles. There is evidence to suggest that the infection spreads insidiously through the cell-to-cell spread infection mechanisms and therefore, there is no rapid dissemination of the virus. This is consistent with the observed absence of mature viral particles in the infected human brains [8,11]. The cytopatologic effects of NiV infection on the neuronal cells and how virus replication is regulated in these cells are still unknown. In the present study, we used two-dimensional polyacrylamide gel electrophoresis (2D-PAGE) and mass spectrometry (MS) to examine the human neuronal cell protein responses to NiV infection.

## Results

### Comparison of 2D-PAGE protein profiles of NiV-infected SK-N-MC cells

**Figure 1:**
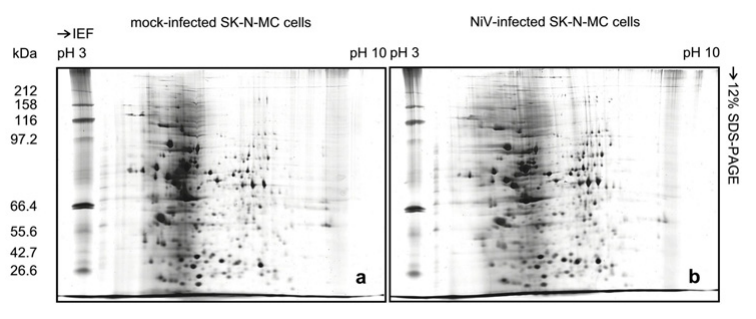
**Two-dimensional gel electrophoresis of mock-infected and NiV-infected SK-N-MC cells**. Mock-infected and NiV-infected cell proteins were extracted directly using urea buffer. IEF was performed in 7 cm IPG strips, pH 3–10 using 100 μg of mock-infected (a) and NiV-infected (b) SK-N-MC cell proteins.

**Figure 2:**
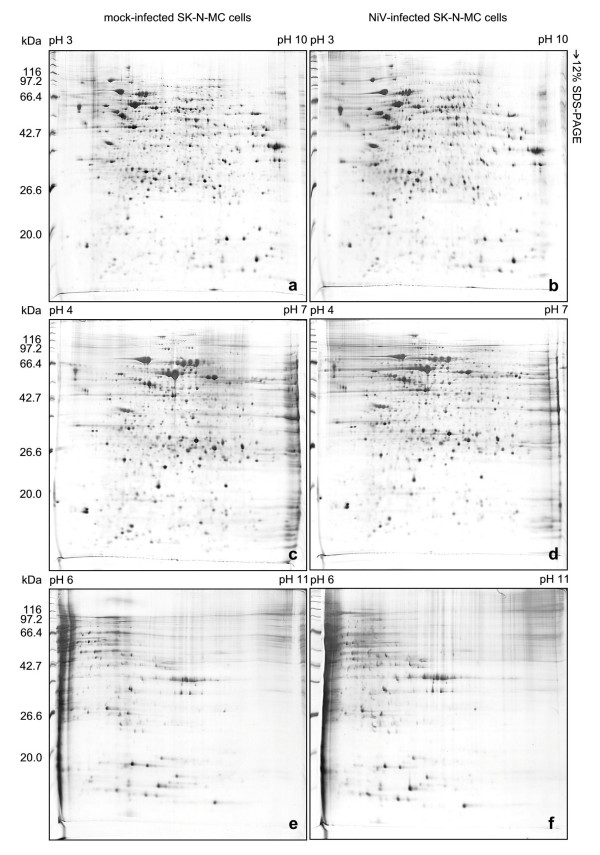
**Enhancement of protein spot separation of mock-infected and NiV-infected SK-N-MC cells for two-dimensional gel electrophoresis analysis**. Improved protein resolution for mock-infected and NiV-infected cell proteins was achieved using the 18 cm IPG strips of pH 3–10 (a, b), pH 4–7 (c, d) and pH 6–11 (e, f), respectively.

The NiV-infected and mock-infected human neuronal cells (SK-N-MC) 2D-PAGE protein profiles were established using four sets of immobilized pH-gradient (IPG) strips: broad (pH 3–10, 7 cm and 18 cm) and narrow range strips (pH 4–7, 18 cm and pH 6–11, 18 cm). At least 397 and 403 protein spots were detected in the silver-stained 2D-PAGE gels of the NiV-infected and mock-infected SK-N-MC cells, respectively (Figures 1a and 1b) using the short IPG strips (7 cm) and the small polyacrylamide gel electrophoresis (PAGE) format (7 cm) to separate the protein extracts. Protein spots between the molecular mass of approximately 97 kDa to 43 kDa, however, were poorly resolved. Improved protein spot separation was achieved using the longer IPG strips (18 cm) and larger PAGE format (18 cm) with more than 1000 protein spots detected using the broad range IPG strip, pH 3–10 (Figures 2a and 2b). However, several clusters of unresolved protein spots were still noted. For analytical purposes, these highly saturated protein spots present between pH 4 to 8 were resolved using the narrower range IPG strips, pH 4–7 and pH 6–11 (Figures 2c, d, e and 2f).

**Figure 3:**
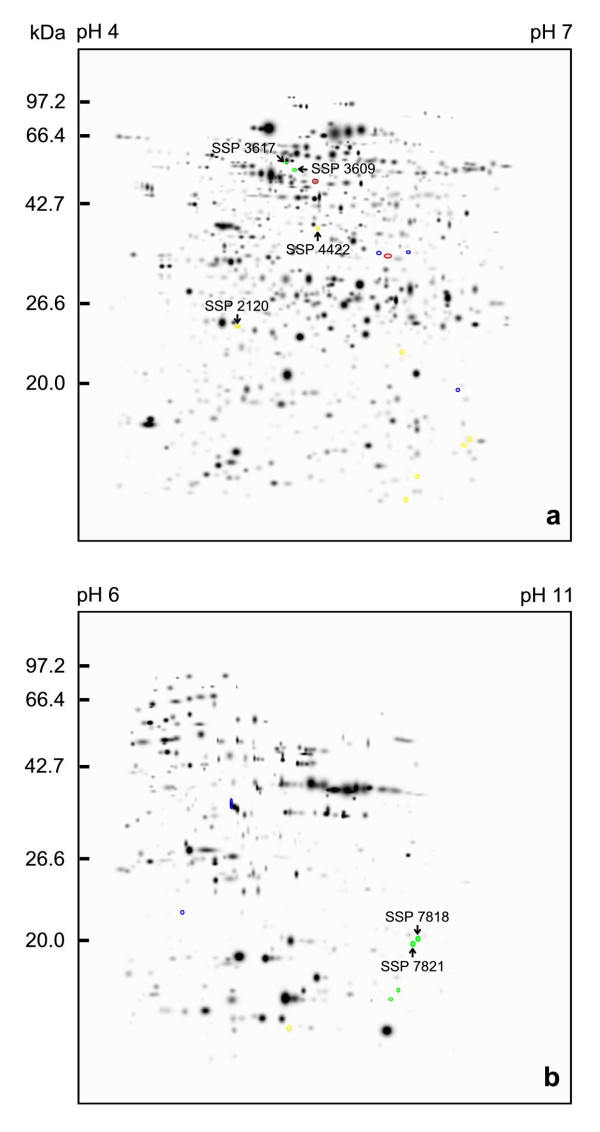
**Composite gel images of the 2D-PAGE protein pattern profiles of SK-N-MC cells before and after NiV infection**. Mock-infected and NiV-infected SK-N-MC cell proteins on 18 cm IPG strips of pH 4–7 (a) and pH 6–11 (b) were analyzed using The Discovery Series PDQUEST 2-D analysis software version 7.2.0 (Bio-Rad Laboratories, USA). Protein spots unique to NiV-infected cells are circled in blue and protein spots absent in the NiV-infected cells are in red. The differentially expressed proteins are circled in green and yellow, indicating spots that are either over abundant (up-regulated) or under represented (down-regulated), respectively. The protein spots were labeled with their unique identification numbers.

A total of 804 protein spots each were visualized in the NiV-infected and mock-infected SK-N-MC cells protein profiles, respectively, using the pH 4–7 large format gels (Figures 2c and 2d). In the pH 6–11 large format gels of the NiV-infected and mock-infected SK-N-MC cells protein profiles, at least 372 and 370 protein spots were detected, respectively (Figures 2e and 2f). A standard reference gel image for each pH range was then constructed from the 2D-PAGE of the mock-infected SK-N-MC cell proteins. Gel image analysis was performed by comparing the occurrence of every spot among the two sets of protein profiles (NiV-infected and mock-infected SK-N-MC cell proteins, each consisting of three gels) against the respective standard gel of the same pH range. Following the detection analysis, unique protein spots, protein spots present only in NiV-infected or mock-infected SK-N-MC cell protein profiles, were detected. At least three protein spots were found to be unique in the pH 4–7 gels of the NiV-infected SK-N-MC cell samples and two in the mock-infected samples (Figure 3a). In the pH 6–11 gels, two unique protein spots were detected in the NiV-infected SK-N-MC cell protein profile (Figure 3b). Several differentially expressed protein spots were detected in the pH 4–7 protein profiles of the NiV-infected and mock-infected SK-N-MC cells. At least two protein spots were over-abundant (up-regulated) in the infected SK-N-MC cell protein profile (Figure 3a) and seven protein spots were markedly under represented (down-regulated). In the pH 6–11 protein profiles of the NiV-infected SK-N-MC cells, four protein spots were up-regulated and one was down-regulated (Figure 3b).

### Identification of proteins by MALDI-TOF MS

**Figure 4:**
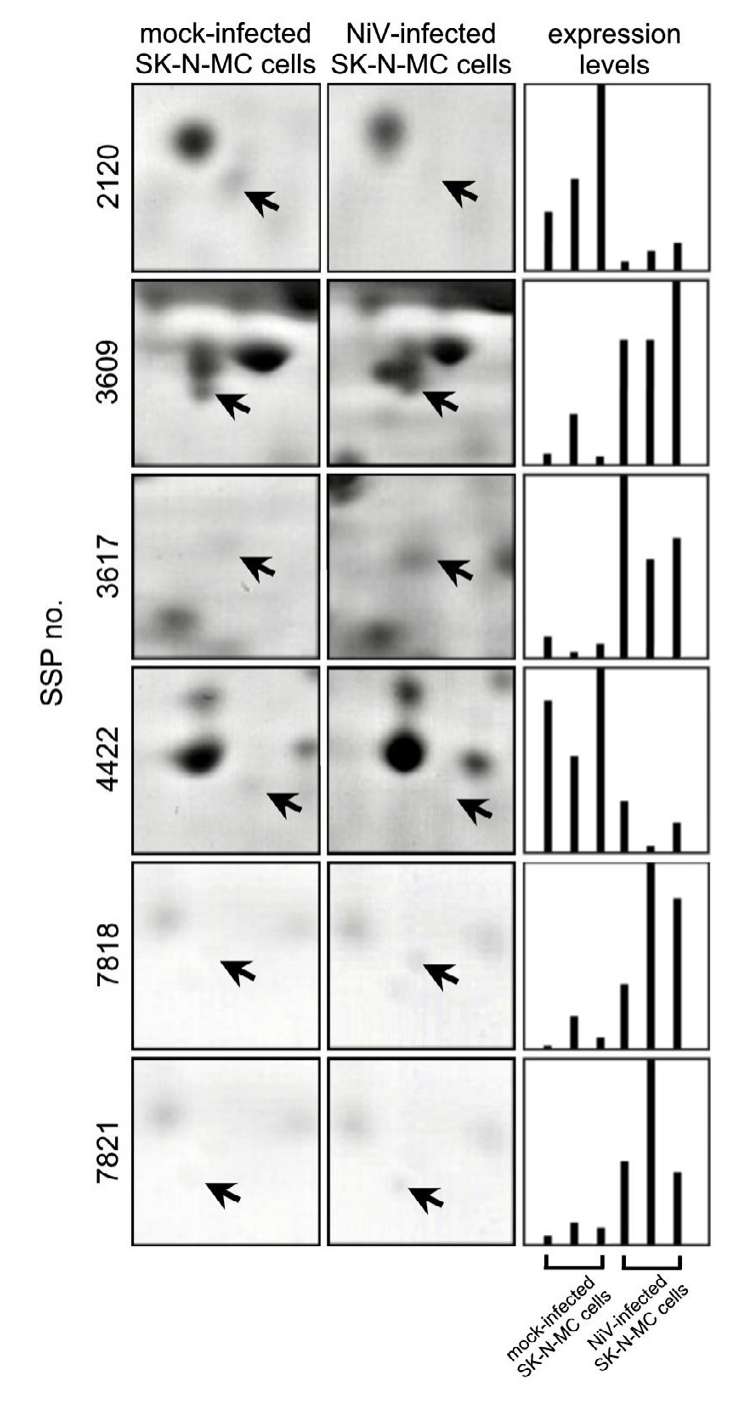
**Differential expression profiles of selected SK-N-MC cell proteins before and after NiV infection**. The representative protein spots showed their increased or decreased in expression (arrow) in the mock-infected and NiV-infected SK-N-MC cells. The differential expression levels of the protein spots upon NiV infection are noted from their relative ratios of protein spot intensity.

The 21 protein spots identified to be either unique or differentially expressed were excised from the 2D-PAGE and subjected to MALDI-TOF MS analysis. Highly interpretable MS spectra with strong MALDI signals was obtained for seven protein spots from the NiV-infected and mock-infected cell protein profiles but only six protein spots were successfully identified with high confident matches using the peptide mass finger printing (PMF) database search (Table 1). Sequence coverage of at least 23% and probability score of 72 were obtained for each of these protein spots. At least seven peptides were found to accurately match the respective proteins in the PMF identification. Ubiquinol-cytochrome-c reductase complex core protein 1 (cytochrome bc1) (Figure 4, SSP no. 3609), heterogeneous nuclear ribonucleoprotein (hnRNP) F (Figure 4, SSP no. 3617), voltage-dependent anion channel 2 (VDAC2) (Figure 4, SSP no. 7818) and the guanine nucleotide binding protein (G protein) (Figure 4, SSP no. 7821) were found in abundance in the NiV-infected SK-N-MC cell protein profiles. Conversely, hnRNP H (Figure 4, SSP no. 4422) and hnRNP H2 (Figure 4, SSP no. 2120) were among the protein spots identified to be markedly down-regulated in the NiV-infected SK-N-MC cell protein profiles. The proteins identified, the hnRNPs F, H and H2 are cellular proteins that could be associated with virus replication or RNA synthesis. The other two proteins, VDAC2 and cytochrome bc1, are proteins associated with the mitochondria, whereas, the G protein is known to be involved in the cell signaling pathways. The identity of three of the six proteins, cytochrome bc1, hnRNP F and VDAC2 was further confirmed using MS/MS analysis (Table 2). The identity of the remaining protein spots could not be determined from the MS analysis due to low abundance of the protein in the 2D-PAGE gels.

**Table 1:** Differentially expressed SK-N-MC human neuronal cell proteins following NiV infection identified from MALDI-TOF MS analysis.

SSP no.	Accession no.	Protein Description	Mass in kDa (experiment/predicted)	pI (experiment/predicted)	Sequence coverage (%)	Number of peptides matched	Mowse score	Error (ppm)
pH 4–7								
2120	6065880	Heterogeneous nuclear ribonucleoprotein H2	38.43/49.52	3.96/5.89	46	15	171	14
3609	515634	Ubiquinol-cytochrome-c reductase complex core protein I, mitochondrial precursor	54.55/52.75	5.30/5.94	46	15	170	17
3617	4826760	Heterogeneous nuclear ribonucleoprotein F	57.18/45.87	5.25/5.38	31	8	88	9
4422	57093855	Similar to heterogeneous nuclear ribonucleoprotein H (hnRNP H)	38.53/46.38	4.46/6.61	43	12	131	6
pH 6–11								
7818	8574295	Voltage-dependent anion channel 2	20.00/31.65	9.43/7.49	41	8	88	18
7821	21619296	Guanine nucleotide binding protein (G protein), beta polypeptide 2-like 1	20.00/34.95	9.40/8.37	23	7	72	9

**Table 2:** Differentially expressed SK-N-MC human neuronal cell proteins following NiV infection identified from MALDI-TOF MS/MS analysis

SSP no.	Accession no.	Protein Description	Peptide sequence matched	Number of fragment ions matched	Ion score	Error (ppm)
3609	515634	Ubiquinol-cytochrome-c reductase complex core protein I, mitochondrial precursor	R.NALVSHLDGTTPVCEDIGR.S	12	56	413
3617	4826760	Heterogeneous nuclear ribonucleoprotein F	K.ATENDIYNFFSPLNPVR.V	24	66	89
7818	8574295	Voltage-dependent anion channel 2	K.VNNSSLIGVGYTQTLRPGVK.L	32	12	2587

### Detection of apoptosis in NiV-infected SK-N-MC cells

**Figure 5:**
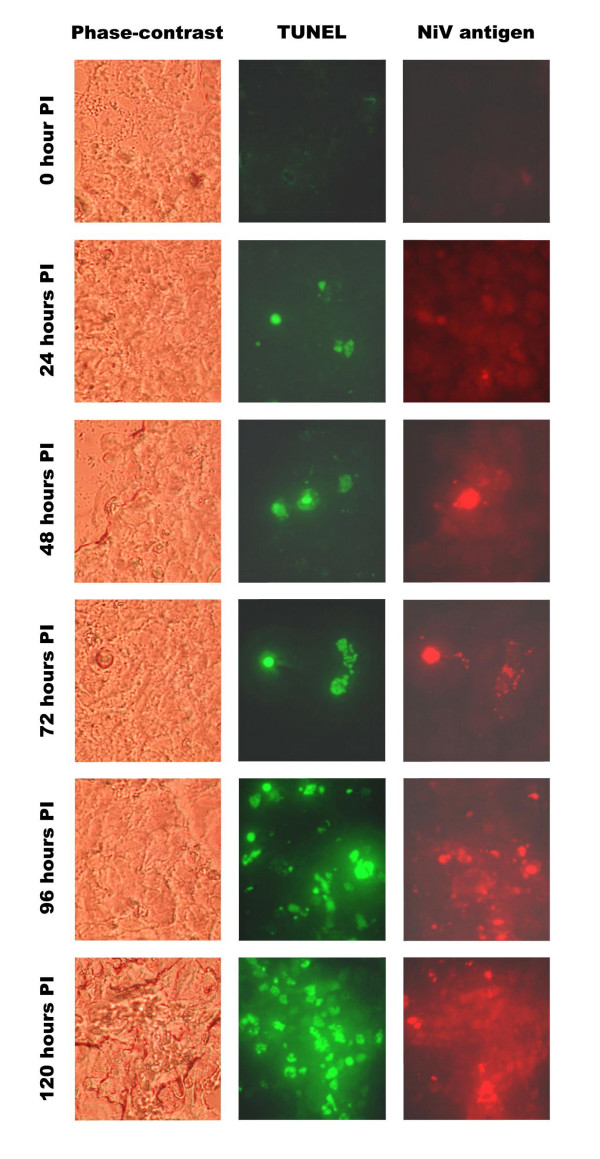
**Detection of apoptosis in NiV-infected SK-N-MC cells**. Mock-infected and NiV-infected SK-N-MC cells were stained with TUNEL and counterstained with the 13A5 NiV monoclonal antibody. The cells were observed under a UV-equipped microscope (63X) at 24 hr, 48 h, 72 h, 96 hr and 120 hr PI. Apoptotic and the NiV-infected positive cells stained fluorescent green and red, respectively.

The abundant presence of the mitochondrial-associated proteins along with the ultrastructural changes to the mitochondria in the NiV-infected neuronal cells raised the possibility of induction of apoptosis. Using terminal deoxynucleotidyl transferase (TdT)-mediated dUTP nick-end labeling (TUNEL) system, apoptotic NiV-infected SK-N-MC cells were detected in the infected cell cultures beginning at 24 hours post-infection (PI) (Figure 5). The number of apoptotic cells steadily increased thereafter and by 96 hours PI, almost the entire cell monolayer became apoptotic. The intensity of the fluorescing cells also increased as the infection progressed. The presence of NiV antigens in these cells was demonstrated using immunofluorescence staining with monoclonal antibody specific against NiV.

## Discussion

NiV infection causes significant cellular morphological changes in the CNS of humans [8]. Infected cells are usually enlarged and giant multinucleated syncytial cells are common [8,12]. NiV infects cells through ephrin-B2, a common cell surface molecule found especially in neuronal cells [13]. NiV virions are released by budding from the infected cells [11] and high number of extracellular virions is obtained towards the terminal end of the infection [12,14]. The rate of progression of the cytopathologic effects of NV infection in human neuronal cells, as well as the intracellular and extracellular virus RNA synthesis are relatively low in comparison to the fully susceptible human fibroblast cells or pig kidney cells [12]. Additionally, the production and peak level of NiV release from the neuronal cells are also lower as compared to the other two NiV-infected cell cultures. These suggest that for reasons that are still unknown, NiV replicates less efficiently in neuronal cells despite having high ephrin-B2 on its surface to facilitate NiV entry. One possible mechanism is through specific cellular factors present in the different cell types.

In the present study, we examine the human neuronal cell protein responses to NiV infection and compare it to that of the mock-treated cells. The focus on neuronal cells is to help in understanding the reasons why NiV is not as efficiently replicated in this cell, whilst the infection is perhaps that caused the severe to fatal infection in humans. Total protein comparison is made using cellular proteins separated by the 2D-PAGE. The 2D-PAGE protein profile enabled direct comparison of the differentially expressed proteins between infected and non-infected samples. Moreover, using bioinformatics application, the differences in protein profile can be pin-pointed and the level of significance in expression can be quantitatively estimated. The method for separation of the NiV-infected and mock-infected SK-N-MC human neuronal cell proteins, and the 2D-PAGE protein profiles are described for the first time here. The number of proteins resolved by the 2D-PAGE across the different pI ranges is consistently reproducible and representative of the total number of proteins resolvable using the 2D-PAGE. At least 800 protein spots were used for the comparative analysis and each consensus gel is built from at least triplicate gels. Though sufficient number of proteins are resolved by the 2D-PAGE, there are possibly many other cellular proteins that are missed as these proteins are either inherently difficult to resolve such as the highly basic proteins and some membrane bound proteins, or they are present in very low abundance that is beyond the detection limit of the silver staining used in the 2D-PAGE. In spite of these limitations, invaluable information is still possible from the analysis of the abundantly expressed proteins in the standardized 2D-PAGE gels from the NiV-infected SK-N-MC cells.

The six significant differentially expressed proteins confidently identified using MS and MS/MS are important cellular proteins associated with various cell functions. The hnRNPs in particular are involved in the regulation of RNA synthesis of both cells and virus RNAs, and influence mRNA processing, trafficking, and stability [15,16]. The hnRNPs H and H2 found suppressed in NiV-infected cells bind to a guanine-rich sequence in pre-mRNAs, downstream of the polyadenine [poly(A)] addition site, and activate or influence the efficiency of pre-mRNA processing [17]. The binding of H and H2 is affected by hnRNP F, found in abundance in NiV-infected SK-N-MC cells. The hnRNP F binds to the same sequence region as the hnRNPs H and H2 but it blocks the binding of the cleavage stimulatory factor 74 kDa subunit that results in the inhibition of the cleavage-polyadenylation reaction [18,19]. The abundance of hnRNP F perhaps results in inhibition of polyadenylation of NiV mRNAs in neuronal cells infection [20,21] and this may have affected the efficiency of NiV replication resulting in the low number of NiV released from infection of the human neuronal cells [12]. As the expression levels of hnRNP F and hnRNPs H and H2 is differentially regulated in pairs [18,22], the findings from the present study could reflect the importance of the hnRNP F/hnRNP H and H2 ratio in the regulation of neuronal cell responses to NiV infection and replication. We also found that the G protein and the mitochondria associated proteins, VDAC2 and cytochrome bc1 are significantly increased in the NiV-infected human neuronal cells. The specific roles of these proteins in NiV infection are presently unknown. The G protein, however, is usually peripherally associated with the plasma membrane and plays important role in the signal transduction mechanism. One possible association between the increase in G protein and NiV infection is perhaps related to binding of NiV to ephrin-B2, a protein highly expressed in the neurons [13] that acts as receptor for NiV [23,24] and activation of the G protein signaling pathways [25]. It is possible that increased expression of the G protein is to compensate for the lost of the G protein function following binding of NiV to ephrin-B2. Alternatively, the abundance of this protein in NiV infection could be important in controlling the infection, perhaps by modulating cellular responses to the infection through the Src-kinase and mitogen-activated protein kinase mediated pathways [26,27]. The mitochondrial proteins VDAC2 and cytochrome bc1 found in abundance in NiV-infected human neuronal cells, on the other hand, are two proteins that could be associated with the induction of apoptosis and cellular pathologic response to the infection. Increase in VDAC2, a mitochondrial porin family [28] may contribute to the increase in the permeability and subsequently, causes the swelling of the mitochondrial matrix observed previously in NiV infected cells [12]. This can lead to the rupture of the mitochondrial outer membrane and release of the mitochondrial proapoptotic factors [29]. These factors then induce apoptosis to the neuronal cell cultures seen in the present study. Increased abundance of cytochrome bc1, a component of the ubiquinol-cytochrome c reductase complex (cytochrome bc1 complex) in NiV infection, on the other hand, is perhaps to help sustain the cytochrome bc1 complex/mitochondrial-associated activities as a consequent to the dysfunction of the mitochondrial respiratory chain or electron transport, or in providing extra energy required to support enhanced protein synthesis, particularly the proteins for virus replication and virus production [30]. While these are all possible, further investigation is required as the cytochrome bc1 complex is also associated with other cell functions including signal transduction and cytokine induction of intercellular adhesion molecule 1 (ICAM-1) expression [31,32].

## Conclusion

Our findings in this study identify the human neuronal cell proteins that are differentially expressed following NiV infection. This represents the first study using proteomic technologies that determine and identify cellular protein modifications in the course of NiV infection. The proteins identified are associated with various cellular functions and their abundance reflects the potential significance in the cytopathologic responses to the infection and the regulation of NiV replication. Whether these proteins differentiate human neuronal cells against the cellular responses of other highly susceptible cells to NiV infection remain to be investigated. Thus, future studies shall focus on the specific roles of each protein, in particular the role of hnRNPs and their relevance in the development of antiviral strategies against NiV and other henipaviruses.

## Methods

### Cells and virus

SK-N-MC cells obtained from ATCC (USA) were maintained in Eagle's minimum essential medium (EMEM from Flowlab, Australia) supplemented with 10% fetal calf serum (FCS, BioWhittaker, Belgium), 2 mM of glutamine, 0.1 mM of non-essential amino acids, 1 mM of sodium pyruvate, penicillin (100 U/mL) and streptomycin (100 μg/mL) at 37°C in 5% CO2. Pig NiV isolate, NV/MY/99/VRI-2794 maintained as previously described [14] was used. This NiV isolate is 99.9% identical to the reported human NiV isolates that is most likely to have been transmitted to humans through direct contact with infected pigs [7]. Throughout the study, adherent SK-N-MC cells were infected with NiV to give an estimated multiplicity of infection (MOI) of 0.2 per cell. Cells treated with mock-infection fluid were prepared in parallel to be used as mock-infection controls. All the treatments were done minimally in triplicates and all research activities that involve the handling of infectious virus were performed in a biosafety laboratory level 3 (BSL-3) facility at the Veterinary Research Institute, Perak, Malaysia.

### Protein sample preparation

NiV-infected and mock-infected cells were harvested for proteins at 72 hours PI. Cells were sedimented by centrifugation at 1,000 × g for 10 minutes and the pellet was lysed in lysis buffer [40 mM Tris, 4% 3-[(3-cholamidopropyl)-dimethylammonio]-1-propanesulfonate (CHAPS), 0.2% bio-lyte 3/10, 8 M urea, 2 mM tributylphosphine(TBP)]. The suspension was then sonicated for 15 minutes using a Branson Sonifier 250 (Branson Ultrasonic, USA) and endonuclease was added to a final concentration of 0.2 unit/μL. After the incubation, the respective cell lysate was pooled and centrifuged at 40,000 × g for one hour and the protein supernatant was collected. Protein concentration was determined using the Micro BCA™ Protein Assay System (Pierce Biotechnology, USA).

### 2D-PAGE

Protein samples (100 μg) was diluted in rehydrating buffer containing 8 M urea, 2% 3- [(3-cholamidopropyl)-dimethylammonio]-2-hydroxy-1-propanesulfonate (CHAPSO), 30 mM dithiothreitol (DTT), 0.5% IPG buffer of pH 3–10 and 0.0007% bromophenol blue and applied to 7 cm IPG strips of pH 3–10. A total of ~300 μg of protein samples were used for the 18 cm, pH 3-10 IPG strips and ~600 μg of protein samples were used for the pH 4–7 and 6–11 strips. The IPG strips were rehydrated with the protein sample mixture at 50 V for 12 hours at 20°C on the Ettan IPGphor IEF System (GE Healthcare, USA). The proteins were then separated by isoelectric focusing (IEF) using the following parameters with current limit of 50 μA/strip: 200 V for 200 V/hour, 500 V for 500 V/hour and 1,000 V for 1,000 V/hour at gradient mode, and 4,000 V for 16,000 V/hour at step and hold mode. Triplicates of the rehydrated 18 cm IPG strips were separated using similar parameters with the exception of the final step that included separation at 8,000 V for 32,000 V/hour for pH 3–10 and 8,000 V for 36,000 V/hour for pH 4–7 and 6–11. After IEF, the strips were subjected to two-step equilibration in equilibration buffers containing 6 M urea, 375 mM Tris-HCl, pH 8.8, 2% sodium dodecyl sulfate (SDS) and 25% glycerol with 65 mM DTT for the first step, and 260 mM iodoacetamide for the second step. The IPG strips were then electrophoresed on 12% SDS- PAGE gel at a constant current for 15 mA for 1 hour, 17.5 mA for 1 hour and finally 20 mA for 5 hours per gel. The analytical and preparative gels were stained with silver stain [33] or colloidal Coomassie Brilliant Blue [34], respectively. Digital images of the analytical gels were acquired and analyzed quantitatively for differentially expressed proteins using The Discovery Series PDQUEST 2-D analysis software version 7.2.0 (Bio-Rad Laboratories, USA). The level of significance of the differences was calculated using the Student's t-test at 95% significance level.

### Mass spectrometric analysis

Protein spots from the triplicate gels were excised from the 2D-PAGE gels using the Ettan™ Spot Picker (GE Healthcare, USA) and transferred to the Ettan™ Spot Handling Workstation (GE Healthcare, USA) for handling of protein gel plugs. The gel plugs were destained in 50% methanol containing 50 mM ammonium bicarbonate. The gel plugs were then digested with trypsin for two hours at 37°C at a final concentration of 0.02 μg/μL of trypsin (Sequencing Grade Modified Trypsin, Promega, USA) in 20 mM ammonium bicarbonate. Peptides were extracted from the gel plugs three times using 0.1% trifluoroacetic acid (TFA) and 50% acetonitrile (ACN). The solvent was then evaporated at 37°C and the dried peptides were reconstituted in 0.5% TFA and 50% ACN. The peptides were spotted onto MALDI-TOF sample slides together with the saturated α-cyano-4-hydroxy cinnamic acid matrix (LaserBio Labs, France) prepared in 0.5% TFA and 50% ACN. Tryptic peptide mass spectra were then obtained using the Voyager-DE™ STR MALDI-TOF workstation MS (Applied Biosystems, USA). PMF search was performed using several available web search engines: MASCOT [35], ProFound [36] and MS-Fit [37]. Searches were performed mainly against databases for Mammalia, Homo sapiens or limited to Viruses with the following parameters: carboxymethylation of cysteine, oxidation of methionine, one missed cleavage, peptide mass tolerance at 50 ppm and monoisotopic masses. Confidence in a given match was based on: (1) the percentage of matching peptide coverage versus the size of the matched protein; (2) the number of matched peptides versus the number of searched peptides; (3) the probability-based MOWSE Score obtained for the matched protein and (4) the error associated with the matched peptides for each sequence [38]. Subsequently, MS/MS analysis was performed using the two most abundant ions obtained in the PMF mass spectra. MS/MS ion search was performed using the MASCOT MS/MS data search [35]. Searches were performed against databases and search parameters as mentioned above with the additional parameter of MS/MS mass tolerance at 0.4 Da.

### Detection of apoptotic cells

NiV-infected cell cultures were stained for apoptosis using the TUNEL system (Promega, USA) following strictly to the manufacturer's protocol. Following TUNEL staining, the infected cells were also stained for NiV antigen using the 13A5 NiV monoclonal antibody [39], followed by TRITC-conjugated goat anti mouse IgG. All the stained samples were viewed under a UV-equipped microscope (Axiolab; Zeiss, Germany) and images were captured using a Digital SLR Camera (Nikon D70, Nikon, Japan).

## Competing interests

The author(s) declare that they have no competing interests.

## Authors' contributions

The corresponding author, Sazaly AbuBakar is the principal investigator of the study, was involved in the design, supervision, data analyses and writing of the report. Li-Yen Chang performed all the laboratory experiments, analyses of data and writing of the report. A.R. Mohd Ali contributed in the virological investigations. Sharifah Syed Hassan was involved in the virological investigations and supervision for the usage of the BSL-3 facility. All authors have read and approved the final manuscript.

## References

[B1] Mayo MA (2002). A summary of taxonomic changes recently approved by ICTV. Arch Virol.

[B2] Mayo MA (2002). Virus Taxonomy – Houston 2002. Arch Virol.

[B3] Anonymous (1999). Outbreak of Hendra-like virus – Malaysia and Singapore, 1998–1999. MMWR Morb Mortal Wkly Rep.

[B4] Anonymous (1999). Update: Outbreak of Nipah virus – Malaysia and Singapore, 1999. MMWR Morb Mortal Wkly Rep.

[B5] Tan KS, Tan CT, Goh KJ (1999). Epidemiological aspects of Nipah virus infection. Neurol J Southeast Asia.

[B6] Goh KJ, Tan CT, Chew NK, Tan PSK, Kamarulzaman A, Sarji SA, Wong KT, Abdullah BJJ, Chua KB, Lam SK (2000). Clinical features of Nipah virus encephalitis among pig farmers in Malaysia. N Engl J Med.

[B7] AbuBakar S, Chang LY, MohdAli AR, Sharifah SH, Yusoff K, Zamrod Z (2004). Isolation and molecular identification of Nipah virus strains from pigs. Emerg Infect Dis.

[B8] Hooper P, Zaki S, Daniels P, Middleton D (2001). Comparative pathology of the diseases caused by Hendra and Nipah viruses. Microbes Infect.

[B9] Wong KT, Shieh WJ, Zaki SR, Tan CT (2002). Nipah virus infection, an emerging paramyxoviral zoonosis. Springer Semin Immunopathol.

[B10] Hyatt AD, Zaki SR, Goldsmith CS, Wise TG, Hengstberger SG (2001). Ultrastructure of Hendra virus and Nipah virus within cultured cells and host animals. Microbes Infect.

[B11] Goldsmith CS, Whistler T, Rollin PE, Ksiazek TG, Rota PA, Bellini WJ, Daszak P, Wong KT, Shieh WJ, Zaki SR (2003). Elucidation of Nipah virus morphogenesis and replication using ultrastructural and molecular approaches. Virus Res.

[B12] Chang LY, Mohd Ali AR, Sharifah SH, AbuBakar S (2006). Nipah virus RNA synthesis in cultured pig and human cells. J Med Virol.

[B13] Hafner C, Schmitz G, Meyer S, Bataille F, Hau P, Langmann T, Dietmaier W, Landthaler M, Vogt T (2004). Differential gene expression of Eph receptors and ephrins in benign human tissues and cancers. Clin Chem.

[B14] Chang LY, Mohd Ali AR, Sharifah SH, AbuBakar S (2006). Quantitative estimation of Nipah virus replication kinetics in vitro. Virol J.

[B15] Birney E, Kumar S, Krainer AR (1993). Analysis of the RNA-recognition motif and RS and RGG domains: conservation in metazoan pre-mRNA splicing factors. Nuclei Acids Res.

[B16] Krecic AM, Swanson MS (1999). HnRNP complexes: composition, structure, and function. Curr Opin Cell Biol.

[B17] Arhin GK, Boots M, Bagga PS, Milcarek C, Wilusz J (2002). Downstream sequence elements with different affinities for the hnRNP H/H' protein influence the processing efficiency of mammalian polyadenylation signals. Nucleic Acids Res.

[B18] Alkan SA, Martincic K, Milcarek C (2006). The hnRNPs F and H2 bind to similar sequences to influence gene expression. Biochem J.

[B19] Veraldi KL, Arhin GK, Martincic K, Chung-Ganster LH, Wilusz J, Milcarek C (2001). hnRNP F influences binding of a 64-kilodalton subunit of cleavage stimulating factor to mRNA precursors in mouse B cells. Mol Cell Biol.

[B20] Fogel BL, McNally MT (2000). A cellular protein, hnRNP H, binds to the negative regulator of splicing element from Rous sarcoma virus. J Biol Chem.

[B21] Jacquene S, Mèreau A, Bilodeau PS, Damier L, Stoltzfus CM, Branlant C (2001). A second exon splicing silencer within human immunodeficiency virus type 1 tat exon 2 represses splicing of Tat mRNA and binds protein hnRNP H. J Biol Chem.

[B22] Honoré B, Baandrup U, Vorum H (2004). Heterogeneous nuclear ribonucleoproteins F and H/H' show differential expression in normal and selected cancer tissues. Exp Cell Res.

[B23] Bonaparte MI, Dimitrov AS, Bossart KN, Crameri G, Mungall BA, Bishop KA, Choudhry V, Dimitrov DS, Wang LF, Eaton BT, Broder CC (2005). Ephrin-B2 ligand is a functional receptor for Hendra virus and Nipah virus. Proc Natl Acad Sci USA.

[B24] Negrete OA, Levroney EL, Aguilar HC, Bertolotti-Ciarlet A, Nazarian R, Tajyar S, Lee B (2005). EphrinB2 is the entry receptor for Nipah virus, an emergent deadly paramyxovirus. Nature.

[B25] Lu Q, Sun EE, Klein RS, Flanagan JG (2001). Ephrin-B reverse signaling is mediated by a novel PDZ-RGS protein and selectively inhibits G protein-coupled chemoattraction. Cell.

[B26] Roberts DJ, Waelbroeck M (2004). G protein activation by G protein coupled receptors: ternary complex formation or catalyzed reaction?. Biochem Pharmacol.

[B27] Lwa SH, Chen WN (2005). Hepatitis B virus X protein interacts with β5 subunit of heterotrimeric guanine nucleotide binding protein. Virol J.

[B28] Sorgato MC, Moran O (1993). Channels in mitochondrial membranes: knowns, unknowns, and prospects for the future. Crit Ret Biochem Mol Biol.

[B29] Shoshan-Barmatz V, Israelson A, Brdiczka D, Sheu SS (2006). The voltage-dependent anion channel (VDAC): function in intracellular signaling, cell life and cell death. Curr Pharm Des.

[B30] Gómez-Puertas P, Albo C, Pérez-Pastrana E, Vivo A, Portela A (2000). Influenza virus matrix protein is the major driving force in virus budding. J Virol.

[B31] Schreck R, Rieber P, Baeuerie PA (1991). Reactive oxygen intermediates as apparently widely used messengers in the activation of the NF-kappa B transcription factor and HIV-1. EMBO J.

[B32] Arai T, Kelly SA, Brengman ML, Takano M, Smith EH, Goldschmidt-Clermont PJ, Bulkley GB (1998). Ambient but not incremental oxidant generation effects intercellular adhesion molecule 1 induction by tumour necrosis factor α in endothelium. Biochem J.

[B33] Blum H, Beier H, Gross HJ (1987). Improved silver staining of plant proteins, RNA and DNA in polyacrylamide gels. Electrophoresis.

[B34] Neuhoff V, Arold N, Taube D, Ehrhardt W (1988). Improved staining of proteins in polyacrylamide gels including isoelectric focusing gels with clear background at nanogram sensitivity using Coomassie Brilliant Blue G-250 and R-250. Electrophoresis.

[B35] MASCOT. http://www.matrixscience.com/search_form_select.html.

[B36] ProFound. http://www.unb.br/cbsp/paginiciais/profound.htm.

[B37] MS-Fit. http://prospector.ucsf.edu/prospector/4.0.8/html/msfit.htm.

[B38] Alfonso P, Rivera J, Hernáez B, Alonso C, Escribano JM (2004). Identification of cellular proteins modified in response to African swine fever virus infection by proteomics. Proteomics.

[B39] Imada T, Abdul Rahman MA, Kashiwazaki Y, Tanimura N, Syed Hassan S, Jamaluddin A (2004). Production and characterization of monoclonal antibodies against formalin-inactivated Nipah virus isolated from the lungs of a pig. J Vet Med Sci.

